# Prognostic Value of Oxylipins for the Development of ESKD

**DOI:** 10.34067/KID.0000000000000347

**Published:** 2024-01-25

**Authors:** Richard J. Roman

**Affiliations:** Department of Pharmacology, University of Mississippi Medical Center, Jackson, Mississippi

**Keywords:** oxylipins, kidney disease, lipidomics and aging

ESKD affects more than 800,000 patients in the United States, and 35 million suffer from CKD. The Medicare cost for the treatment of ESKD now exceeds 50 billion dollars/year,^1^ which is 20 times the current budget of National Institute of Diabetes and Digestive and Kidney Diseases, and the cost for CKD is 150 billion/yr. Despite the widespread use of angiotensin converting enzyme inhibitors and angiotensin II receptor blockers, along with the more recent introduction of sodium-glucose cotransporter 2 inhibitors, aldosterone, and glucagon-like peptide agonists that also slow the progression of CKD,^2^ the number of patients with ESKD is still increasing, and it parallels the epidemic of obesity and diabetes in our aging population. Thus, there is a tremendous need for better prognostic biomarkers for the risk of ESKD in patients who could benefit from prophylactic renoprotective intervention. The recent completion of the 1 million patient genome-wide association study and many others has identified numerous single nucleotide polymorphisms in candidate genes associated with CKD.^3^ Despite the recent advancements in identifying genetic abnormalities linked to CKD, there is still a significant need for further expansion in this area, particularly concerning the validation of causal mutations and subsequent implementation of genetic tests to assess the risk of developing ESKD.

The current paper by Surapaneni *et al.*^4^ in this issue of *Kidney360* provides evidence that assessing plasma oxylipin levels may provide a new set of biomarkers to help address the perplexing problem of determining a patient's risk for ESKD. These investigators used a novel liquid chromatography/mass spectroscopy/mass spectroscopy lipidomic approach to potentially measure the concentrations of >1100 metabolites of arachidonic acid and other fatty acids in plasma samples collected between 1990 and 1993 from 9650 older patients with normal renal function in the Atherosclerosis Risk in Communities Study. These patients were followed for 30 years to see if they developed ESKD. They successfully identified 223 plasma lipids in these patients. The plasma levels of 11-hydroxy-9-octadecenoic acid and a dihydroxydocosapentaenoic acid correlated with a reduced risk for ESKD, while arachidonic acid was correlated with an increased risk. They performed a GWAS in 8406 of these same patients who were genotyped and identified four genomic regions correlated with the levels of these metabolites. A subsequent transcriptome-wide analysis nominated several differentially expressed genes associated with lipid metabolism within 500 Kb of GWAS loci linked to the metabolite levels. The results suggested that stearoyl-CoA desaturase expression in adipose tissue and polycystin 2 levels in the brain were associated with the rs603424 locus on chromosome 10 linked to hydroxyoctadecenoic acid levels in this population. Acyl-CoA thioesterase 2 expressions in blood and UDP glucuronosyltransferase 2 expression in the liver were strongly associated with regions on chromosomes 14 and 4 linked with dihydroxydocosapentaenoic acid levels. The expression of fatty acid desaturase 1 was correlated with the rs28456 locus on Chrm 11 associated with plasma arachidonic acid levels. However, little is known about the regulation of 11-hydroxy-9-octadecenoic acid, dihydroxydocosapentaenoic acid, and arachidonic acid levels, so additional studies are needed to determine how the genetically linked loci and nearby differentially expressed genes might be involved.

Very little is known about the role of oxylipins derived from fatty acids other than arachidonic acid on renal function and the pathogenesis of CKD. Dietary supplementation of omega-3 polyunsaturated fatty acids may reduce acute renal injury by enhancing proresolvin anti-inflammatory metabolites. Previous studies have reported that mutations in cytochrome P450 enzymes that produce 20-hydroxyeicosatetraenoic acid (20-HETE) and epoxyeicosatrienoic (EET) and dihydroxyeicosatrienoic (DiHETE) acids are associated with the development of hypertension, stroke, or obesity. Other studies found that knockout of genes involved in the metabolism of arachidonic acid to prostaglandins, leukotrienes, 20-HETE, EETs, and DiHETEs alter BP and renal injury.^5,6^ Changes in the urinary excretion of 20-HETE are associated with diabetic nephropathy in Black patients.^7^ Two recent studies have used global plasma lipidomics to identify other CKD-associated lipids.^8,9^ However, this study is the first to identify novel oxylipin biomarkers correlated with ESKD risk. Much more work is needed to understand the mechanisms involved since little is known about the metabolism of 11-hydroxy-9-octadecenoic acid and dihydroxydocosapentaenoic acid or their potential effects on renal function and inflammation in CKD. Arachidonic acid is the rate-limiting substrate for the formation of prostaglandins, leukotrienes, EETs, DiHETEs, and 20-HETE. These eicosanoids have all been implicated in cardiovascular and kidney disease, so genetic or dietary elevations in arachidonic acid levels might alter the production of one of these downstream mediators. However, it is remarkable how a single measurement of plasma oxylipin levels could have predicted these patients' lifetime risk for the development of ESKD. Perhaps the oxylipin levels reflect underlying genetic abnormalities in lipid metabolism or dietary preferences that favor the formation of proinflammatory versus anti-inflammatory oxylipins that eventually led to CKD and ESKD. Additional studies that replicate these findings and longitudinally profile the long-term changes in the levels of oxylipins in at-risk patients are crucial. The paper is noteworthy as it is the first comprehensive profiling of plasma oxylipin levels associated with kidney disease. This same analytical approach will likely successfully identify lipid biomarkers associated with cardiovascular disease, arteriosclerosis, Alzheimer disease, diabetic nephropathy, and other forms of kidney disease.
